# Neuromuscular control in males and females 1 year after an anterior cruciate ligament rupture or reconstruction during stair descent and artificial tibial translation

**DOI:** 10.1038/s41598-023-42491-6

**Published:** 2023-09-15

**Authors:** Angela Blasimann, Aglaja Busch, Philipp Henle, Sven Bruhn, Dirk Vissers, Heiner Baur

**Affiliations:** 1 Division of Physiotherapy, School of Health Professions, Bern University of Applied Sciences, Bern, Switzerland; 2 Department of Rehabilitation Sciences and Physiotherapy, Faculty of Medicine and Health Sciences, University of Antwerp, Wilrijk, Belgium; 3 University Outpatient Clinic, Sports Medicine & Sports Orthopaedics, University of Potsdam, Potsdam, Germany; 4 Sonnenhof Orthopaedic Center, Lindenhof Group AG, Bern, Switzerland; 5 Department of Orthopaedic Surgery and Traumatology, Inselspital, Bern University Hospital, University of Bern, Bern, Switzerland; 6 Institute of Sports Science, University of Rostock, Rostock, Germany

**Keywords:** Anatomy, Diseases, Health care, Medical research, Risk factors, Signs and symptoms

## Abstract

Neuromuscular alterations are reported in patients with anterior cruciate ligament reconstruction (ACL-R) and conservative treatment (copers with ACL deficiency, ACL-C). However, it is unclear whether sex influences neuromuscular control. The objective was to investigate differences in neuromuscular control regarding sex and treatment type one year after ACL rupture in comparison to a group with an intact ACL (ACL-I). Electromyography of vastus medialis (VM) and lateralis, biceps femoris (BF) and semitendinosus (ST) was recorded in ACL-R (N = 38), ACL-C (N = 26), and ACL-I (N = 38) during stair descent and reflex activity by anterior tibial translation while standing. The movements of stair descent were divided into pre-activity, weight-acceptance and push-off phases, reflex activity in pre-activation, short, medium (MLR), and long latency responses (LLR). Normalized root mean squares for each muscle of involved and matched control limb per phase were calculated and analyzed with two-way ANOVA (α = 0.05). During stair descent, neuromuscular differences of BF were significant during push-off only (p = 0.001). Males of ACL-R and ACL-C had higher BF activity compared to ACL-I (p = 0.009, 0.007 respectively). During reflex activity, VM and BF were significantly different between treatment groups for pre-activation (p = 0.013, 0.035 respectively). VM pre-activation of females was higher in ACL-R compared to ACL-C (p = 0.018), and lower in ACL-C compared to ACL-I (p = 0.034). Males of ACL-R showed higher VM and less BF pre-activation (p = 0.025, p = 0.003 respectively) compared to ACL-I. Males of ACL-C had less BF pre-activation compared to ACL-I (p = 0.019). During MLR, intra-group differences in ST were found for treatment (p = 0.011) and females of ACL-R compared to ACL-I (p = 0.015). During LLR, overall intra-group differences in VM were present for treatment (p = 0.034) and in females (ACL-R versus ACL-C (p = 0.015), ACL-I (p = 0.049), respectively). One year after an ACL rupture, neuromuscular alterations persist regardless of treatment and sex. Standard rehabilitation protocols may not be able to restore neuromuscular control. Future research should include long-term follow up and focus on exercises targeting neuromuscular function.

## Introduction

Neuromuscular control includes volitional and reflexive activity before and during static and dynamic tasks, as it has been stated for a neuromuscular training to enhance unconscious motor responses by stimulating afferent signals and central mechanisms responsible for dynamic joint control^1^. Adequate neuromuscular control leads to active knee stability during controlled, known activities of daily living (ADL) but also in situations with sudden (tibial) perturbations. In these cases, neuromuscular control does not only consist of adequate neuromuscular answers after initial foot-floor contact but also appropriate pre-activation of knee stabilizing muscles before landing. Neuromuscular control during static and dynamic tasks can be directly assessed by electromyography (EMG), of which a wide range of outcome measures has been reported in the literature^2^.

After a rupture of the anterior cruciate ligament (ACL), altered sensorimotor control and neuromuscular adaptations^3,4^, due to the loss of the mechanoreceptors of the native (original) ACL leading to altered afferent inputs to the central nervous system^5,6^, have been reported. Patients with an ACL are treated either non-surgically (conservatively with rehabilitation alone) or surgically with different graft types for ACL reconstruction or suture^7^. A 5-year follow-up did not reveal any differences in patient-reported and objective outcomes between ACL patients with early reconstruction, conservative treatment followed by reconstruction or rehabilitation alone^8^. However, neuromuscular alterations—in terms of increased hamstrings activation in the reconstructed leg compared to the contralateral leg—were found in physically active people after ACL reconstruction during functional activities such as (stair) walking and stepdown tasks^9–12^ or in athletes during sport-specific assessments such as landing after a hop^11,13^. Similar results were also reported in ACL deficient patients with conservative treatment during walking^14^, step tasks^15^, level walking and jogging^16^.

Clearance for return to sport (RTS) after ACL rupture is mainly based on clinical examinations, patient-reported outcome scores and widely used physical performance test batteries^17–19^, despite a wide range of EMG outcome measures for neuromuscular control reported in the literature^2^. Moreover, no consensus exists about gold standard for safe RTS assessments and cut-off points^20–22^. After RTS, failure rates of the graft up to 19% and contralateral ACL rupture postoperatively in up to 24% of the cases were reported^23–25^. The high rates of re-injury and secondary ACL rupture, and a lack of validity of RTS criteria after ACL reconstruction are inacceptable and indicate that more research is needed^21,22,26^.

Therefore, the purpose of this cross-sectional study was to investigate neuromuscular activity in participants one year after an ACL rupture or reconstruction, at the timepoint of unrestricted clearance for RTS after completion of rehabilitation. The study compared conservatively and surgically treated participants, females and males, during stair descent and artificially induced tibial translation while standing, in comparison to a healthy control group. Based on literature^10–12,27,28^, it is hypothesized that, because of altered afferent information, the neuromuscular quadriceps response will be downregulated and hamstring EMG activity will be upregulated in patient cohorts compared to controls. Subjects with former ACL injury may have developed different neuromuscular strategies than subjects who have never torn their ACL^3^. Therefore, it is hypothesized that neuromuscular control will still be impaired in conservatively (ACL-C) and surgically treated participants (ACL-R) one year after ACL rupture compared to healthy controls. Due to the controversial findings regarding sex-specific differences in neuromuscular control^29^, no hypothesis regarding subgroups of females and males are made.

## Methods

A cross-sectional, experimental study design with two patient groups and a healthy control group was determined to investigate differences of neuromuscular control related to treatment for females and males one year after an ACL reconstruction or rehabilitation alone.

### Sample size calculation

An a priori analysis of effect size and sample size was made for a desired power of 95% and an α-error of 0.05 (software G*Power, version 3.1, Heinrich Heine University of Duesseldorf, D)^30^, based on own pilot data^9,27^. Considering the variability of data, an a priori necessary sample size for independent comparisons of n = 10 (α = 0.05, actual power: 0.96, effect size d: 1.78) was set. To account for dropouts and subgroup analysis (treatment modality and sex as dependent samples), a total number of n = 15 females and males per group was planned to be recruited.

### Participants

Persons could be included if they were between 16 and 60 years old, with unequivocally detectable, unilateral ACL status, physically active at least 2×/week for 45 min, and had a Tegner activity score of 5 or higher^31–33^. Magnetic resonance imaging was available in all participants with former ACL rupture. Patients could be included with any type of ACL reconstruction except dynamic intraligamentary stabilization (Ligamys, Mathys AG, Bettlach, CH) and similar techniques. Time since rupture, reconstruction respectively, had to be between 11 and 14 months. Status of ACL was clinically examined in all participants to allow for group allocation. This clinical examination included testing of active and passive range of motion of both the femorotibial and patellofemoral joint, Lachman test in 20° knee flexion, Anterior Drawer Test in 90° knee flexion and Pivot Shift Test by always the same experienced sports physical therapist. General exclusion criteria were former knee pathology before ACL rupture, other injury of the lower extremity, back pain, musculoskeletal disorders refraining from test protocol, cardiac, neurologic, or peripheral vascular disease, acute infection, alcohol abuse, current pain medication, thrombosis, pregnancy, dementia, and not being able to understand written or oral German.

**Figure 1:**
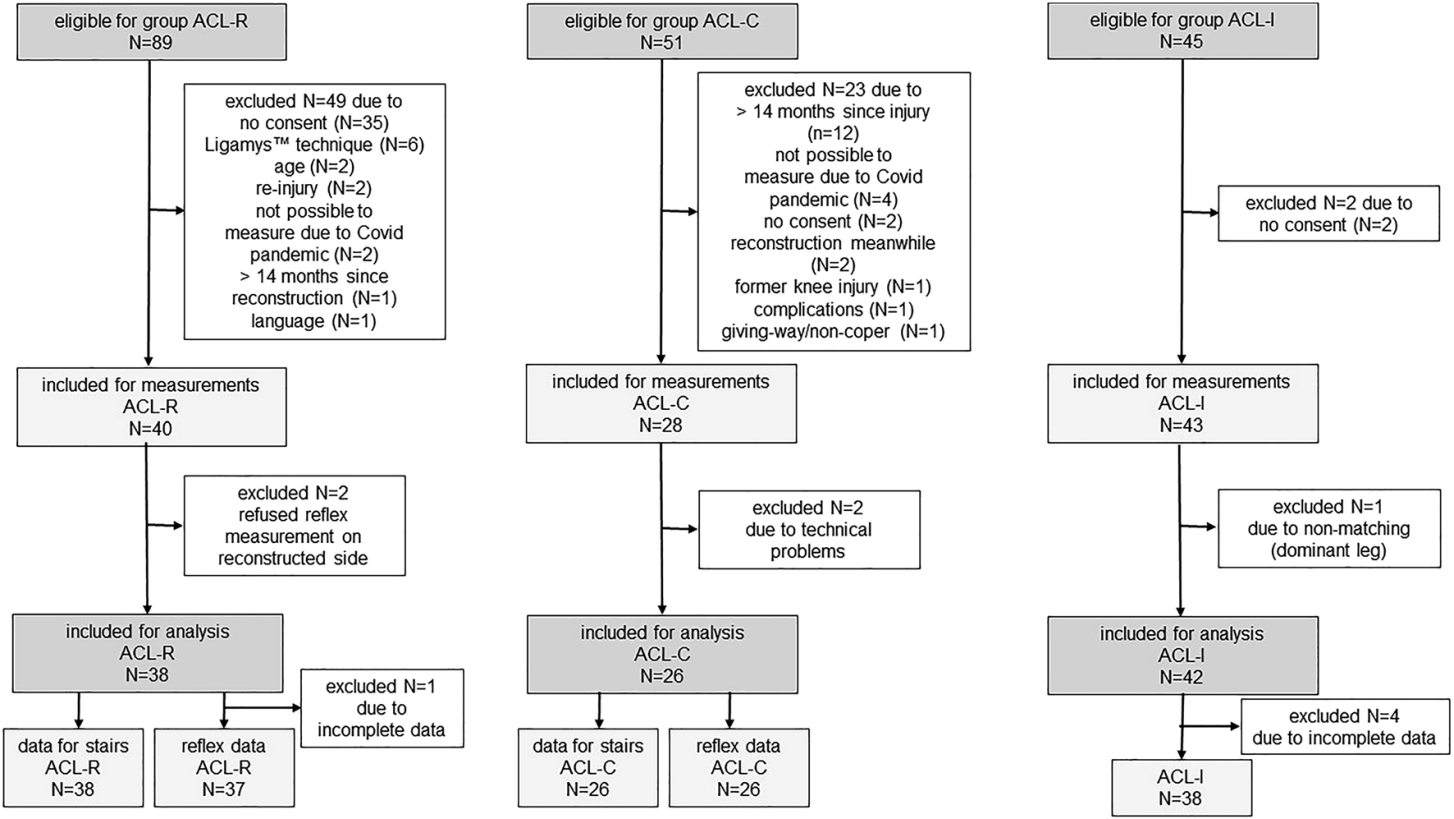
Flowchart describing number of participants during recruitment process and data sets analysed. ACL-R, ACL reconstructed group; ACL-C, ACL group with conservative treatment; ACL-I, healthy control group with intact ACL; N, number of.

Participants for the ACL-R and ACL-C group were mainly recruited through the Sonnenhof Orthopaedic Center, Lindenhof Group AG, Bern (Switzerland) by an orthopaedic surgeon between January 2018 and August 2021. In addition, surgically and conservatively treated patients were recruited through private physiotherapy practices, sports clubs, and private networks during the same period. Information was mainly spread by physiobern (official professional body of physiotherapists in the Cantone of Berne, Switzerland) through newsletters for members, social media, but also via word-of-mouth recommendation. The control group consisted of healthy people with an anamnestically intact ACL (ACL-I) who had been recruited from local sport clubs, and among members of the Bern University of Applied Sciences (Switzerland). Individual group matching for initially two control groups was based on sex, age, body height, body mass and dominant leg, defined as the preferred leg to kick a ball with^31,32^. For data analysis, the two matched control groups were combined to one, later named ACL-I with N = 38 healthy participants. In total, N = 185 participants volunteered for this study and were assigned to one of three groups, based on ACL status (Fig. 1).

### Measurements

All measurements took place at the Bern Movement Lab (Bern University of Applied Sciences, Bern, Switzerland) by using a setup described in former publications^9,10,27^.

Anthropometric data (age, height, body mass), limb dominance as well as data regarding physical activity such as type of sports, number of hours per week and Tegner activity score^33^ were collected with a standardized case report form (CRF). Moreover, patient-reported outcomes regarding pain, other symptoms, activities of daily life, sports and knee-related quality of life were assessed with the Knee injury and Osteoarthritis Outcome Score (KOOS)^34^. Afterwards, the participants were prepared for the EMG measurements. The skin was shaved, smoothed, and cleaned with alcohol to prepare for bipolar, self-adhesive electrodes (Ambu BlueSensor, Ambu A/S, Ballerup, DK; Type P-00-S, inter-electrode distance: 20 mm) being applied on the vastus medialis (VM), vastus lateralis (VL), semitendinosus (ST) and biceps femoris (BF) muscle on both limbs according to SENIAM standards^35^. The inter-electrode impedance was controlled (D175 Electrode Impedance Meter, Digitimer, Herfordshire, UK) and accepted ≤ 2 kΩ. The reference electrode was placed on the right patella. Before the measurements started, the actual wellbeing and pain level of the participants were assessed by using a visual analogue scale (VAS)^36^.

**Figure 2:**
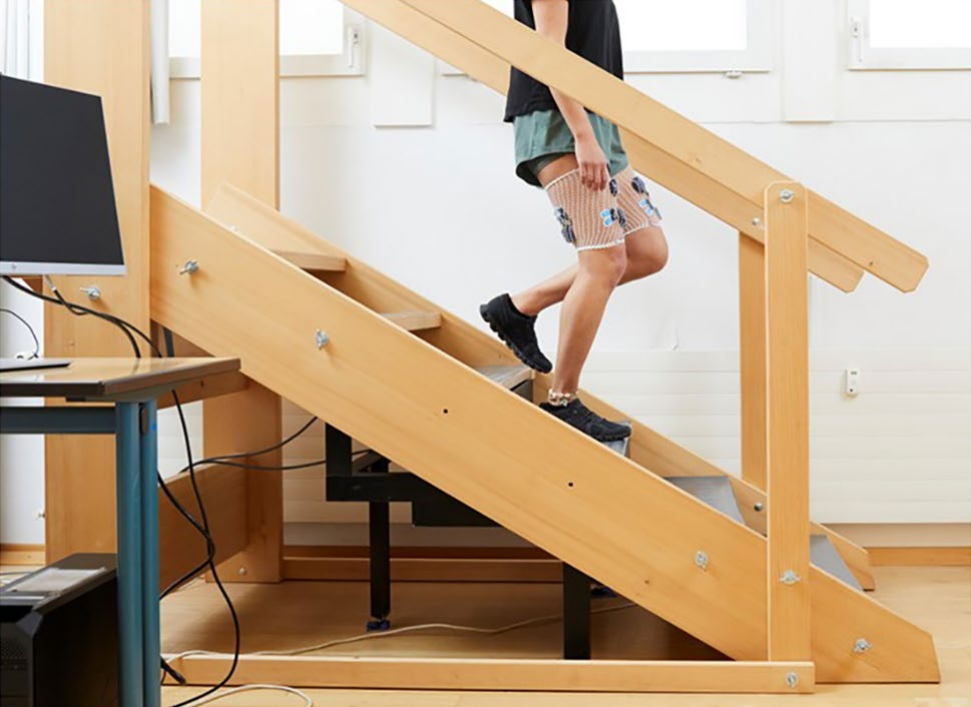
Functional task: walking downstairs on custom-made wooden stairway (©BFH Bern Movement Lab).

**Figure 3:**
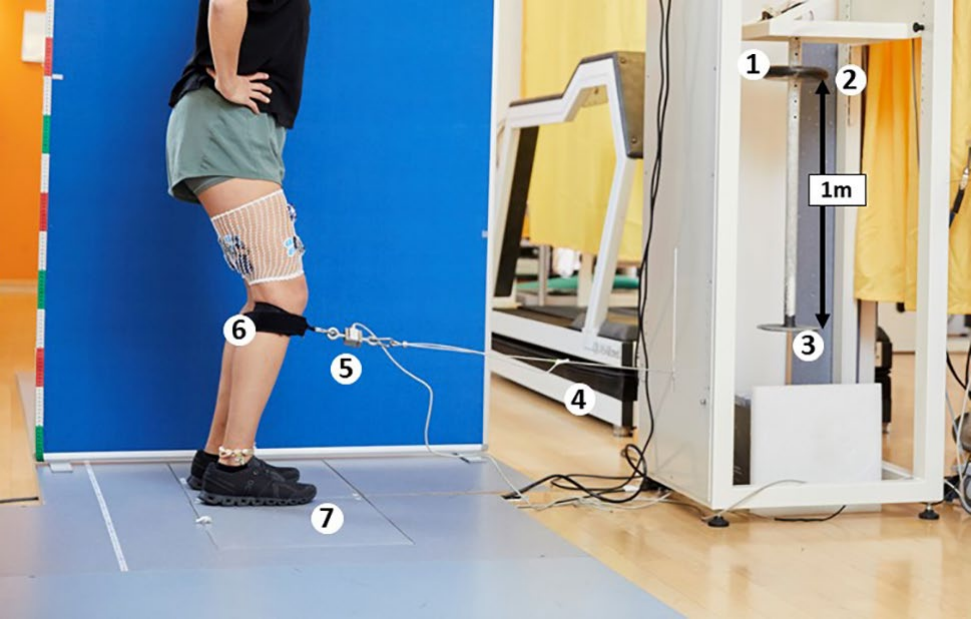
Stretch reflex measurements in upright standing position (©BFH Bern Movement Lab). (1) Electromagnet, (2) falling barbell weight, (3) stopper, (4) wire rope, (5) force transducer, (6) brace, (7) two force plates.

Each participant started with a warm-up on a treadmill (quasar med, h/p/cosmos sports & medical GmbH, Nussdorf-Traunstein, D) for 10 min at 1.39 m/s (5 km/h). Initial contact of each gait cycle was detected by two force transducers (type KMB52, 10kN, MEGATRON Elektronik GmbH & Co. KG, Putzbrunn, D) mounted under the treadmill. Surface electromyography (EMG) signals of VM, VL, BF and ST were recorded during treadmill walking at the above-mentioned speed for 2 min. These signals were used for submaximal EMG normalization^37,38^. Following this warm-up, each participant completed two experimental situations in the same order: stair descent (Fig. 2) and stretch reflex measurements induced by artificial tibial translation in posterior-anterior direction (Fig. 3).

#### Stair descent as functional task

Participants descended a six-step stairway ten times at self-selected speed without using the handrails. The custom-made wooden stairway (Fig. 2) with an inclination of 30.6°, a step height of 17.1 cm and a step depth of 29.0 cm^39^ had two embedded multicomponent force plates (type 9286BA, Kistler Instrumente AG, Winterthur, CH) in the third and fourth step identifying gait cycles during stair climbing.

Movement cycles of stair descent were divided into pre-activation (PRE), weight acceptance (WA) and push-off (PO) phases. The PRE phase included 150 ms prior to initial foot-floor contact until initial contact. The WA phase was defined from initial contact to the lowest applied vertical ground reaction force (equal to “braking phase” until anterior–posterior force crosses the zero line). After the WA phase, the PO phase followed until vertical ground reaction forces decline to zero (“propulsion phase”).

#### Stretch reflex measurements: artificial tibial translation in posterior-anterior direction

The participants were standing upright with arms akimbo and knees slightly bent (30° flexion) (Fig. 3). To guarantee equal distribution of the body mass, participants placed each foot on a force plate (Type 9286BA, Kistler Instrumente AG, Winterthur, CH) and controlled body mass distribution by visual feedback provided by a computer screen at eye level. All participants were blinded to the time point of artificially induced tibial translation. Moreover, participants wore headphones for music and an attenuator to avoid any acoustic anticipation. Both legs were tested in randomized order (www.randomization.com).

A standardized impulse for tibial translation was applied to the tibial shank by a rope and pulley system in posterior-anterior direction^40^, monitored by a force transducer (type KM1506, 2 KN, MEGATRON Elektronik GmbH & Co. KG, Putzbrunn, Germany) (Fig. 3). The impulse was the trigger signal for the onset of tibial translation and was elicited 2 × 15 times per lower extremity. In between each stimulus, a break of about 30 s was performed to avoid impulse anticipation and to allow subjects to adjust the standardized position if necessary. Between the series, a short break allowed the participants to walk around and relax to avoid excessive co-contraction or muscular fatigue. After the measurements, the general wellbeing and actual pain levels of the participants were assessed again as described previously.

### Outcomes

Root mean squares (RMS) values of EMG signals during stair descent in the respective pre-defined time intervals (PRE, WA, PO) were used as outcomes^37,38^. Moreover, RMS values of EMG signals after the activation of the stretch reflex (following onset of tibial translation) were calculated in four pre-defined time intervals: − 50 to 0 ms background activity, pre-activation (PRE_50) respectively, 20–40 ms short latency response (SLR), 40–60 ms medium latency response (MLR) and 60–95 ms long latency response (LLR)^40–43^.

### Signal transmission, data processing and normalization

In general, there was no delay due to synchronization since all systems (force sensor signals from treadmill, rope-pulley system and stairs) were recorded together with the EMG signals in one LabVIEW-based software (IMAGO Record, pfitec, Endingen, D). No later synchronization during the post-processing was needed.

EMG signals were transmitted across a differential-preamplifier to a telemetric main amplifier (PowerPack, pfitec, Endingen, D), band-pass filtered at 10–1000 Hz and recorded at 2000 Hz for treadmill walking and stair descent, and at 4000 Hz for the stretch-reflex measurement^44^. Then, the signals were converted from analogue to digital (type NI PCI 6255, National Instruments, Austin, USA), registered and further processed with the same LabVIEW-based software (IMAGO Record, pfitec, Endingen, D). Afterwards, all raw EMG signals were full wave rectified. Additionally, raw EMG signals from treadmill walking and stair descent were band-pass filtered at 10–500 Hz (Butterworth, 2nd order). RMS values were calculated for the defined time windows as described above.

Afterwards, RMS values were exported in Excel spreadsheets (Windows 10, Microsoft Corporation, Redmond WA, USA), where individual means out of 30 gait cycles (treadmill walking), ten steps (stair descent), and 30 tibial translations per extremity for each muscle in each time interval were calculated. RMS values were normalized according to the corresponding time intervals retrieved during treadmill walking—defined as 100% of neuromuscular activity—and used for inter-subject-comparability. Reflex responses during artificial tibial translation, and activations during stair descent were normalized and expressed as a percentage (% EMG) of respective treadmill walking activity^37,38^. For each normalized RMS, a plausibility check was done based on the mean RMS for each muscle per timeframe. If individual RMS values exceeded two standard deviations (SD) for stair descent and three SD for reflex measurement (due to high intra-individual variability), suspicious values were traced back to the original data set, recalculated and eventually excluded.

### Statistical analysis

Data were transferred from CRFs to Excel spreadsheets (Windows 10, Microsoft Corporation, Redmond WA, USA) and later processed by the Statistical Package for the Social Science (SPSS) software (SPSS Statistics for Windows, version 28.0, IBM, Armonk NY, USA). Participants’ characteristics were checked for normal distribution (Kolmogorov–Smirnov), followed by non-parametric t-tests (Mann–Whitney-U and Kruskal–Wallis) to evaluate age, body height, and weight, physical activity level, Tegner, KOOS, leg dominance, wellbeing, and pain level for significant differences between groups. Additionally, a parametric two-way analysis of variance (ANOVA) for repeated measures with the factors sex and treatment was performed^45^. All EMG outcomes (dependent variables) of all muscles (VM, VL, ST, BF) for the injured leg of ACL-R, ACL-C and the matched ACL-I leg during all movement phases of stair descent (PRE, WA, PO) and the reflex time windows (PRE_50, SLR, MLR, LLR) during artificial tibial translation were analyzed. If data were not normally distributed and heterogeneity of variances was present (Levene test not significant), non-parametric tests for analysis of variance (Kruskal–Wallis-test) were used alternatively. In addition, post-hoc tests (Bonferroni) were applied for the factor group assignment. Effect sizes (ES) were calculated for significant results by using the following equitation (n = total number of participants in the compared groups): $r=\left|\frac{z}{\sqrt{n}}\right|$ with *r* < 0.3 as small effect, 0.3 ≤ *r* < 0.5 as medium effect, and *r* ≥ 0.5 as large effect^46^. The alpha-level was set at 5%.

### Ethics approval and consent to participate

This study was approved by the Ethics Committee of the Canton of Bern (Switzerland), KEK No. 2017-02282, and was conducted in accordance with the Declaration of Helsinki^78^. Written informed consent was obtained from every participant.

## Results

### Characteristics of participants

To allow comparison with other studies, mean values, and standard deviations (SD) are reported although not all data were normally distributed.

There were no significant differences in age (p = 0.419), body height (p = 0.087), body mass (p = 0.507), body mass index (BMI) (p = 0.056), sex (p = 0.419) and leg dominance (p = 0.876) between the groups of ACL-R, ACL-C and ACL-I. However, the three groups differed significantly in physical activity regarding weekly hours (p = 0.014) and level (p =  < 0.0001), which was higher in the patient groups. The mean Tegner activity score of averaged 7 in the ACL-R and ACL-C group indicated a higher level of recreational sports such as alpine skiing, tennis, squash, running or competitive sports like basketball, rugby, ice-hockey, handball, frisbee and Swiss wrestling. Furthermore, also KOOS subscales and total scores (p < 0.0001 all) were significantly higher in ACL-R and ACL-C groups. The groups ACL-R and ACL-C were comparable regarding anthropometric data, time since rupture, weekly hours and level of physical activity (all p > 0.05). More details regarding characteristics of the two patient groups (ACL-R, ACL-C respectively), and healthy controls (ACL-I) are displayed in Table 1 below and in the Tables A.1 & A.2 (supplementary material).

**Table 1:** Characteristics of participants with an ACL reconstruction (ACL-R), with a conservatively treated ACL rupture (ACL-C) and healthy controls with an intact ACL (ACL-I).

Characteristics	Mean ± SD if not otherwise stated			p-value			
	ACL-R N = 38	ACL-C N = 26	ACL-I N = 38	ACL-R vs. ACL-I	ACL-C vs. ACL-I	ACL-R vs. ACL-C	Overall
Age [years]	32.02 ± 12.21	38.38 ± 11.65	33.13 ± 9.16	0.391	0.099	0.031*	0.074
Body height [cm]	173.55 ± 6.25	170.23 ± 7.59	173.66 ± 6.96	0.831	0.033*	0.075	0.087
Body mass [kg]	71.71 ± 11.19	71.06 ± 14.88	68.38 ± 9.43	0.244	0.942	0.456	0.507
BMI [kg/m^2^]	23.94 ± 2.73	24.47 ± 4.64	22.64 ± 2.02	0.015*	0.305	0.305	0.056
Time since injury [months]	12.7 ± 1.4	12.5 ± 1.1	–	< 0.0001*	–	0.579	–
Sex: Ratio of ♀:♂ (%)	17:21 (44.7:55.3)	16:10 (61.5:38.5)	20:18 (52.6:47.4)	0.494	0.848	0.190	0.419
Physical activity [min/week]	425.96 ± 265.10	373.02 ± 158.18	293.26 ± 182.81	0.014*	0.011	0.811	0.014*
Tegner score (max. 10 points)	6.71 ± 1.45	6.96 ± 1.18	5.53 ± 1.31	0.001*	< 0.0001*	0.273	< 0.0001*

If not otherwise stated means, ± standard deviations and p-values are reported. Dashed lines indicate not applicable; * indicates significant p-values (p < 0.05). ACL-R, anterior cruciate ligament reconstructed (= patients); ACL-C, anterior cruciate ligament rupture conservatively treated; ACL-I, anterior cruciate ligament intact (= healthy controls); °Tegner activity score (preinjury) ranging from 0 (sick leave or disability pension) to 10 (competitive sport on a professional level).

### Stair descent

An overview containing mean EMG values, SD and analysis of variance presented for sex and group (treatment option) can be found in Table A.3 (supplementary material).

**Figure 4:**
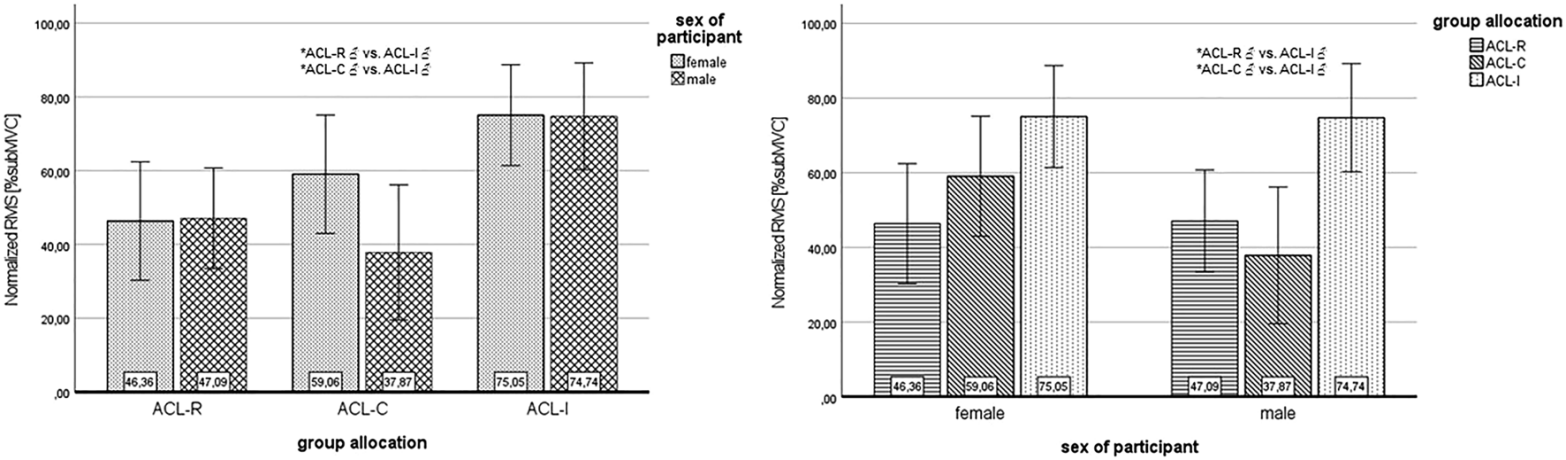
Bar plots of significant results for stair descent. Results are presented for group allocation (left side) and sex (right side); error bars = 95% confidence interval; *significant differences between subgroups (p < 0.05); ACL, anterior cruciate ligament; ACL-C, group with conservative treatment after ACL rupture; ACL-I, healthy controls with intact ACL; ACL-R, group with ACL reconstruction; BF, biceps femoris; CI, confidence interval; involved = formerly injured or reconstructed side, respective matched leg of controls; PO, push-off phase during stair descent; RMS, root mean square values; subMVC, submaximal voluntary contraction (normalized values with treadmill walking = 100% subMVC).

During stair descent, post-hoc analysis comparing neuromuscular activity of the formerly injured, involved leg of ACL-R, ACL-C, and the matched leg of ACL-I (based on side of injury) revealed significant differences of the BF in PO phase only (p = 0.001). Significantly higher neuromuscular activity was found for the BF of ACL-R males in comparison to ACL-I males (p = 0.009, ES 0.43) and of ACL-C males compared to males in the ACL-I group (p = 0.007, ES 0.52). Figure 4 shows bar plots for significant results of BF during PO, provided for group allocation (a) and sex (b). All other comparisons of muscles, phases and group members were not significant (Table A.3, supplementary material).

### Artificial tibial translation

**Figure 5:**
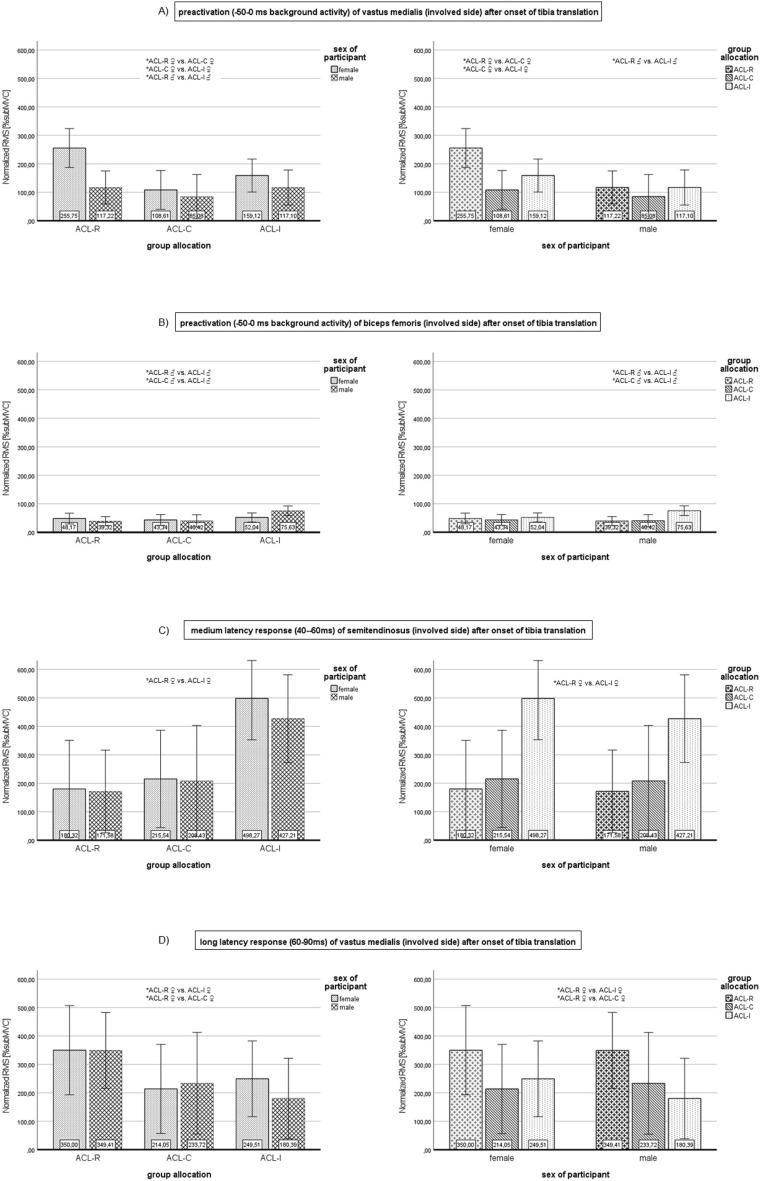
(**A**–**D**): Bar plots of significant results for artificial tibial translation. Results are presented for group allocation (left side) and sex (right side); error bars = 95% confidence interval; *significant differences between subgroups (p < 0.05); ACL, anterior cruciate ligament; ACL-C, group with conservative treatment after ACL rupture; ACL-I, healthy controls with intact ACL; ACL-R, group with ACL reconstruction; BF, biceps femoris; CI, confidence interval involved = formerly injured or reconstructed side, respective matched leg of controls; PRE_50, pre-activation (background activity); MLR, medium latency response; LLR, long latency response; RMS, root mean square values; subMVC, submaximal voluntary contraction (normalized values with treadmill walking = 100% subMVC); VM, vastus medialis; ST, semitendinosus.

Table A.4 (supplementary material) provides an overview of mean EMG values expressed as %RMS, SD, analysis of variance and effect sizes presented for sex and group. Significant results of respective muscles, phases and groups are graphically edited in Fig. 5A–D.

During pre-activation (PRE_50), VM and BF showed overall significant differences comparing ACL-R, ACL-C and ACL-I (p = 0.013, p = 0.035 respectively). Females of ACL-R differed from ACL-C and ACL-I and showed higher RMS values in VM, but only the comparison with females of ACL-C was significant (p = 0.018, ES 0.43) (Fig. 5A). Females of ACL-C differed significantly from ACL-I (p = 0.034, ES 0.36) with lower neuromuscular activity of VM compared to their controls and ACL-R (Fig. 5A). Moreover, males of ACL-R had a significantly higher pre-activation in VM (p = 0.025, ES 0.41) compared to males in ACL-I (Fig. 5A). Regarding BF during PRE_50 only males of ACL-R and ACL-C showed significantly less activity compared to their controls (p = 0.003, ES 0.48; p = 0.019, ES 0.44, respectively) (Fig. 5B). The comparison between male participants after ACL injury was not significantly different (p = 0.860). All additional comparisons between muscles and groups in this phase revealed no significant differences.

No significant comparisons were observed for SLR.

In the time window of MLR, ST showed overall significant results (p = 0.011). Females of ACL-R had significantly lower mean reflex activity compared to the female controls (p = 0.015, ES 0.43) (Fig. 5C). All other comparisons were not significant.

During LLR, significant results were found for VM only (p = 0.034). Neuromuscular activity of VM of ACL-R females was significantly higher than those of ACL-C females (p = 0.015, ES 0.45) but also higher than in females of the control group (p = 0.049, ES 0.34) (Fig. 5D). No significant differences between treatment options or sex were observed for VL or hamstrings during this phase (Table A.4, supplementary material).

More statistical values are provided as supplementary material (Table A.5).

## Discussion

This cross-sectional study investigated neuromuscular activity in patients one year after an ACL rupture with reconstruction or conservative treatment in comparison to a healthy control group. Neuromuscular control was assessed during stair descent as functional task and an artificially induced tibial translation while standing. Beforehand, it was hypothesized that neuromuscular control would still be impaired in conservatively (ACL-C) and surgically treated participants (ACL-R) one year after ACL rupture compared to healthy controls.

In general, we found a large range of differences in activity patterns within and between groups in the investigated timeframes and movement phases indicating a large intra-individual variability, especially during artificial tibial translation. This was in line with further research assessing patients with an acute rupture of the ACL^9^. However, the hypothesis that neuromuscular control would be negatively affected even one year after reconstruction or conservatively treated rupture had been confirmed.

### Stair descent

A significant decrease in neuromuscular activity was found in the BF muscle of male patients during PO in comparison to healthy controls. The altered neuromuscular activity can be interpreted that afferent feedback is still affected one year after ACL rupture or reconstruction, regardless of treatment. At least in conservatively treated patients, this might be a functional adaptation to altered mechanical properties. Obviously, the changes in the brain leading to impaired neuromuscular control in people after ACL rupture may not be sufficiently targeted by current rehabilitation programs which is in line with present findings^47^. During stair descent, Hall and colleagues also found higher hamstrings activity in patients after ACL-R compared to healthy controls^11^. However, the higher activity was found in the medial hamstrings (ST) and the group consisted of males and females with an ACL reconstruction 1–18 years ago^11^. In an early study investigating a one-step climbing and descending task, a significantly earlier onset and longer total duration of lateral hamstrings was observed in copers compared to a healthy control group^15^. Also this group consisted of males and females but was tested one year after ACL injury^15^.

Altered cortical activation after ACL reconstruction had been reported previously: Patients six or more months after ACL rupture showed reorganization of the central nervous system, indicating that an ACL rupture is not only a peripheral musculoskeletal injury but rather a neurophysiologic dysfunction^48^, which is present even years after ACL surgery^3,49^. ACL injury may alter intracortical facilitation^49^ and lead to increased intracortical inhibition. The latter is correlated with decreased capability to voluntarily activate the quadriceps^50^. In our study, the findings regarding quadriceps activation were controversial, and only significant in the following situations and comparisons: females after ACL-R had significantly higher VM activity compared to ACL-C during WA, males of ACL-C showed lower VL activity compared to ACL-I. During PO, males of ACL-R had lower VM and VL activity compared to ACL-C, males of ACL-C had higher VM and VL activity compared to ACL-I, and females of ACL-R had higher VL activity compared to ACL-C.

### Artificial tibial translation

In a similar setting as in the present study, a reduced SLR of hamstrings in combination with increased tibial translation had been reported^42^. However, those measurements had been done after muscle fatigue, indicating a fatigue-related reflex reduction. This reduction could point at the need for more neural “drive” in patients suffering from increased anterior tibial translation caused by ACL rupture, resulting in higher neuromuscular activity to stabilize the knee joint. Therefore, the increased pre-activation in VM and BF as well as reflex activity of the ST during MLR might reflect an active compensation pattern of the hamstrings for higher stability demands. Sufficient reflex response in the MLR timeframe is essential and seems to be better after conservative treatment than after surgical reconstruction when compared to healthy controls in our study. In case of ACL deficiency few weeks after injury, a reduction of MLR was found to be related to “giving-way” symptoms^51^. However, as our participants in the ACL-C group were copers, “giving-way” symptoms had not been assessed previously. In addition, the increased activity during MLR in this group can be seen as compensatory mechanism to guarantee active knee stability.

ST was significantly and highly reduced in female ACL-R compared to female ACL-I, but hamstrings in general tended to be less active in all patient groups. Sex-specific neuromuscular adaptations such as activation timing, differences in force intensity of knee stabilizing muscles^52–57^ and a dominance of the quadriceps over the hamstring muscles in women, which could increase anterior tibial translation, had been reported previously^55,56,58,59^. In contrast, these findings had been contradicted by a systematic review about sex differences in landing and cutting manoeuvres which reported that no proof for quadriceps dominance during the described activities was present^60^.

### Methodological aspects

Stair descent and artificial tibial translation have been selected as representing tasks for important, functionally relevant, and demanding situations.

Stairway walking (ascent and descent) as an ADL activity requires preactivity (joint stiffening) before initial contact to guarantee active joint stability, and eccentric contraction^61^ while 346% of the bodyweight loads the knee joint^62^. Additionally, it has been shown that an anterior–posterior tibial translation occurs during ambulation^63^. Previously published preliminary studies from our research group had shown the feasibility and reliability of this method, and the discriminatory power between different cohorts^39,64^. With this task, which is relevant to everyday life, sensorimotor competence can be assessed in applied situations.

Artificially induced tibial perturbation was chosen as reflex activity and simulation of injury mechanism. Monitoring neuromuscular control during the corresponding time window from pre-activity to perturbation onset and time windows for reflex responses after perturbation give insight into sensorimotor control mechanisms to guarantee knee joint stability^40–42^. During tibial translation in posterior-anterior direction (relatively to the femur), the ACL comes under tension. In healthy subjects with an intact ACL, the hamstring muscles (BF, ST, semimembranosus muscle) act synergistically to this translational movement^40^. This synergistic contraction occurs reflexively during appropriately rapid tibial translation, e.g., by the rope-pulley system triggering sudden tibial perturbations which induce a reflex response of the hamstring muscles^40^. After a sudden, anterior tibial translation, the magnitude of protective reflex activation of the hamstrings results in increased active joint stability of the knee; this mechanism protects against injury^65^. This indicates that the extent of the dynamic neuromuscular joint control of the knee stabilizing muscles can be measured closely to the physiological injury mechanism by surface EMG during standardized tibial perturbations^40,66^.

Eighty percent of all ACL injuries are due to non-contact episodes^67^ while deceleration and acceleration motions with excessive quadriceps contraction or insufficient hamstrings activation at or near full knee extension are seen^68,69^. Thereby, the tibia is translated anteriorly relatively to the femur and stresses the ACL. With an intact ACL, the hamstring muscles act synergistically to this translational movement whereas the quadriceps muscles are ACL antagonist^40^. It has been shown that non-contact ACL ruptures happen 17-50 ms after initial contact^67^, leaving a short time frame for mechanosensory feedback (e.g., reflex response). Pre-activity and reactive neuromuscular responses regulate muscle and joint stiffness, which is influencing dynamic joint stability, consequently influencing ACL injury risk^70^. Monitoring neuromuscular control during exactly this time window from preactivity to onset of perturbation and reflexive time windows after tibial translation give insight into sensorimotor control mechanisms establishing knee joint stability^40–42^.

During artificial tibial translation in standing position, equal distribution of body mass under the right and left foot was monitored. However, if the body mass would shifted more onto the heel or forefoot, the center of mass changed and would lead to more degrees of flexion or extension of the spine, pelvis ante-/retroversion or trunk inclination/reclination^71–73^. With this altered upright starting position, the participants could have unconsciously influenced the activity of the hamstrings which could led to a different neuromuscular strategy due to changes in the length of the superficial back line, a myofascial meridian^74^. Furthermore, the band-sling was placed at the proximal part of the shank over the triceps surae, which could influence the activation of the hamstring muscles due to mechanical stimuli in triceps surae Ia and II afferent pathways^40^.

### Strengths and limitations

This study is one of the few publications reporting neuromuscular activity of thigh muscles comparing two ACL patient groups with different treatment modalities one year after injury. Moreover, to current knowledge, it is the first study which investigated reflex response after artificial induced tibial perturbation in participants with ACL-R or rehabilitation alone (ACL-C). A narrow time frame (11–14 months post-injury, after reconstruction respectively) was chosen for both patient groups where all participants had full clearance for RTS. In addition, the applied methods were standardized and used before. Monitoring neuromuscular control during exactly the time window from pre-activity to perturbation onset and reflexive time windows after joint (stability) perturbation give insight into sensorimotor control mechanisms establishing knee joint stability^40–42^. This method could lead to define outcomes for neuromuscular control to be integrated into current RTS criteria which has already been stated^26,75^. However, the assessment of neuromuscular control as presented in this study is sophisticated but not ready to be used in a clinical setting yet.

Several study limitations should be considered: It was planned a priori to recruit 30 participants per group, 15 females and 15 males. However, we could include fewer participants in the ACL-C group despite the extended recruitment phase. This was mainly due to Covid pandemic, during which the movement laboratory had to be closed for several weeks. By the time the laboratory was accessible again, six subjects—among which four for the ACL-C group—were outside the time window of 14 months post-injury and could no longer be included. As sex seems to affect neuromuscular patterns^41,42,76^, an equal sex distribution in the groups was aimed but could not be reached.

The ACL-R group was heterogenous regarding choice of graft and surgical techniques as participants from more than one orthopaedic surgeon had been included. This led to different treatment modalities regarding duration and quality of rehabilitation as rehabilitation after ACL rupture or ACL reconstruction is not standardized. Moreover, no specialization in sports physical therapy for physiotherapists is needed to treat ACL patients which sometimes negatively affects quality of rehabilitation. Although all included patients were cleared for unrestricted RTS, their physical condition and psychological readiness to participate in sport varied widely when they were measured at the lab: Some participants of the patient groups showed atrophy in the operated leg, experienced painful episodes, had limited knee flexion, were hypermobile in one or both knee joints, or had not returned to the sport level before ACL rupture. Furthermore, the subjects had very different levels of sport, ranging from recreational activities to competitive sport at a national level. Accordingly, the mean EMG values during stair climbing and the reflex activities during tibial translation varied largely which was in line with former research from our group^9,10,27^.

In summary, this heterogeneity in ACL injured participants’ physical and mental state, quality and duration of rehabilitation, experience and training of physical therapist could have influenced our results and limits generalizability of the findings. Nevertheless, the results help to better understand long-term consequences of an ACL rupture, especially for neuromuscular control after RTS^27^.

Another limitation was that we decided to use two-way parametric ANOVA to analyze the factors “group” and “sex” despite non-parametric distribution of most of the variables tested. According to published recommendations^77^, parametric ANOVA may be used in those situations.

Furthermore, adjusted linear models including age, physical activity and BMI as covariates were used to check whether the effects of sex and group changed substantially (> 10% difference in effect sizes). Only the effect of group was substantially different in the adjusted model for the outcome variable “MLR of ST” when age, physical activity and BMI had been included. Both non-adjusted and adjusted effect sizes were still medium, and the effect of confounding was not further investigated.

## Conclusions

The present study revealed that neuromuscular alterations are still present in the involved leg one year after ACL rupture or reconstruction compared to healthy controls. Standard rehabilitation protocols may not be able to restore neuromuscular control. In addition, neuromuscular control seems to be impaired independently from sex after unilateral ACL rupture but influenced by treatment. Therefore, it is important to assess neuromuscular control directly by surface EMG to detect deficits despite fulfilled clinical and physical performance test for RTS.

Future research should aim at more homogenous participant groups, include long-term follow up and standardized rehabilitation programs which focus on exercises targeting neuromuscular function. Another aspect of future research interest are bilateral deficits after ACL rupture to investigate whether the contralateral leg can be an adequate reference for functional evaluation of the injured extremity. As the limb symmetry index is still widely used, but probably underestimating the functional deficits of both lower extremities, other outcome measures for neuromuscular function should be considered when deciding for a safe RTS.

### Supplementary Information

Supplementary Table 1.

Supplementary Table 2.

SupplementaryTable 3.

Supplementary Table 4.

Supplementary Table 5.

## Data Availability

The datasets used and analysed during the current study are available from the corresponding author upon reasonable request and are stored on servers owned by the Bern University of Applied Sciences.
