# Supplementary material for: Neuromuscular control in males and females 1 year after an anterior cruciate ligament rupture or reconstruction during stair descent and artificial tibial translation
Source: Sci Rep. 2023 Sep 15;13:15316. doi: 10.1038/s41598-023-42491-6 (PMC10504317; doi:10.1038/s41598-023-42491-6)
Supplement: Supplementary file 1 — Supplementary Table 1. [file 41598_2023_42491_MOESM1_ESM.docx]

Table A.1: Additional clinical characteristics of participants with an ACL reconstruction (ACL-R), with a conservatively treated ACL rupture (ACL-C) and healthy controls with an intact ACL (ACL-I).

|  | **ACL-R** | **ACL-C** | **ACL-I** | **ACL-R vs. ACL-I** | **ACL-C vs. ACL-I** | **ACL-R vs. ACL-C** | **overall** |
| --- | --- | --- | --- | --- | --- | --- | --- |
| **Characteristics** | **N = 38** | **N = 26** | **N = 38** | **p-value** | **p-value** | **p-value** | **p-value** |
| **Leg dominance (N=)**  right:left [%] | 35:3 (92.1:7.9) | 23:3 (88.5:11.5) | 34:4 (89.5:10.5) | 0.694 | 0.899 | 0.626 | 0.876 |
| **Prehabilitation (N=)**  yes:no [%] | 13:25 (34.2:65.8) | -- | -- | < 0.0001* | -- | < 0.0001* | -- |
| **PT after surgery/injury (N=)**  yes:no [%] | 38:0 (100:0) | 26:0 (100:0) | -- | < 0.0001* | -- | < 0.0001* | -- |
| **KOOS subscale (absolute values)**  pain (9 items, max. 36 p.)  other symptoms (7 items, max. 28 p.)  ADL (17 items, max. 68 p.)  sports & leisure (5 items, max. 20 p.)  HRQoL (4 items, max. 16 p.) | 4.47 ± 3.32  5.95 ± 3.80  2.50 ± 3.42  3.63 ± 2.88  4.73 ± 3.29 | 3.35 ± 2.99  5.08 ± 3.12  2.23 ± 4.03  2.69 ± 2.77  3.81 ± 2.90 | 0.37 ± 0.71  1.58 ± 1.52  0.08 ± 0.36  0.21 ± 0.70  0.37 ± 1.00 | < 0.0001*  < 0.0001*  < 0.0001*  < 0.0001*  < 0.0001* | < 0.0001*  < 0.0001*  < 0.0001*  < 0.0001*  < 0.0001* | 0.119  0.532  0.380  0.151  0.259 | < 0.0001*  < 0.0001*  < 0.0001*  < 0.0001*  < 0.0001* |
| **VAS**  wellbeing pre [mm]  wellbeing post [mm]  pain pre [mm]  pain post [mm] | 5.53 ± 8.60  7.29 ± 9.00  3.08 ± 4.08  6.18 ± 13.25 | 5.35 ± 10.31  5.58 ± 9.91  4.35 ± 8.89  6.42 ± 10.57 | 5.29 ± 6.90  6.55 ± 7.39  1.08 ± 2.06  3.42 ± 7.88 | 0.643  0.937  0.026*  0.264 | 0.666  0.355  0.037*  0.082 | 0.972  0.402  0.977  0.486 | 0.870  0.612  0.045*  0.209 |
| **Medial meniscal tears (N=)**  conservative treatment [%]  suture [%]  resection [%]  none [%] | 2 (5.3)  15 (39.5)  7 (18.4)  14 (36.8) | 4 (15.4)  0  0  22 (84.6) | --  --  --  -- | --  --  --  -- | --  --  --  -- | --  --  --  -- | --  --  --  -- |
| **Lateral meniscal tears (N=)**  conservative treatment [%]  suture [%]  resection [%]  none [%] | 1 (2.6)  8 (21.1)  1 (2.6)  28 (73.7) | 5 (19.2)  0  0  21 (80.8) | --  --  --  -- | --  --  --  -- | --  --  --  -- | --  --  --  -- | --  --  --  -- |
| **Medial collateral ligament injury (N=)**  conservative treatment [%]  surgery [%]  none [%] | 8 (21.1)  1 (2.6)  29 (76.3) | 8 (30.8)  0  18 (69.2) | --  --  -- | --  --  -- | --  --  -- | --  --  -- | --  --  -- |
| **Lateral collateral ligament injury (N=)**  conservative treatment [%]  surgery [%]  none [%] | 2 (5.3)  0  36 (94.7) | 4 (15.4)  0  22 (84.6) | --  --  -- | --  --  -- | --  --  -- | --  --  -- | --  --  -- |
| **Bone bruise (N=)**  yes:no [%] | 0:38 (0:100) | 7:19 (26.9:73.1) | -- | -- | -- | -- | -- |
| **Cartilage defect (N=)**  yes:no [%] | 2:36 (5.3:94.7) | 2:24 (7.7:92.3) | -- | -- | -- | -- | -- |
| **Graft types (N=)**  Quadriceps tendon [%]  Hamstrings tendon [%]  Patellar tendon [%]  Unknown [%] | 26 (68.4)  8 (21.1)  3 (7.9)  1 (2.6) | --  --  --  -- | --  --  --  -- | --  --  --  -- | --  --  --  -- | --  --  --  -- | --  --  --  -- |

Data are presented as mean ± standard deviation (SD) unless otherwise stated. * Indicates significant p-values (p<0.05); dashed lines indicate not applicable.

Legend: ACL-C = anterior cruciate ligament rupture conservatively treated; ACL-I = anterior cruciate ligament intact (= healthy controls); ACL-R = anterior cruciate ligament reconstructed (=patients); ADL = activity of daily life; HRQoL = health-related quality of life; KOOS = Knee injury and Osteoarthritis Outcome Score (scoring per item: 0 = no problems; 4 = extreme problems); max. = maximum; p = points; N= number of; post = after the measurements; pre = before the measurements started; PT = physiotherapy; VAS = visual analogue scale from 0 to 100mm
