# Supplementary material for: Neuromuscular control in males and females 1 year after an anterior cruciate ligament rupture or reconstruction during stair descent and artificial tibial translation
Source: Sci Rep. 2023 Sep 15;13:15316. doi: 10.1038/s41598-023-42491-6 (PMC10504317; doi:10.1038/s41598-023-42491-6)
Supplement: Supplementary file 2 — Supplementary Table 2. [file 41598_2023_42491_MOESM2_ESM.docx]

Table A.2: Characteristics of females and males for all three groups

| **Characteristics** | **ACL-R (N = 38)** | | **ACL-C (N = 26)** | | **ACL-I (N = 38)** | | ACL-R vs. ACL-I | ACL-C vs. ACL-I | ACL-R vs. ACL-C | overall |
| --- | --- | --- | --- | --- | --- | --- | --- | --- | --- | --- |
| **sex** | **females** | **males** | **females** | **males** | **females** | **males** | p-value | p-value | p-value | p-value |
| **number of participants** | **N = 17** | **N = 21** | **N = 16** | **N = 10** | **N = 20** | **N = 18** |  |  |  |  |
| **Age** [years] | 31.71 ± 11.64 | 32.29 ± 12.94 | 39.25 ± 11.48 | 37.00 ± 12.40 | 31.80 ± 9.44 | 34.61 ± 8.87 | 0.391 | 0.099 | 0.031 | 0.419 |
| **Body height** [cm] | 169.12 ± 5.53 | 177.14 ± 4.20 | 165.81 ± 5.39 | 177.30 ± 4.62 | 169.35 ± 6.40 | 178.44 ± 3.70 | 0.831 | 0.033 | 0.075 | 0.087 |
| **Body mass** [kg] | 63.68 ± 8.53 | 78.22 ± 8.61 | 66.76 ± 13.34 | 77.95 ± 15.27 | 62.17 ± 7.82 | 75.29 ± 5.41 | 0.244 | 0.942 | 0.456 | 0.507 |
| **BMI** [kg/m^2^] | 22.4 ± 2.39 | 25.13 ± 2.43 | 24.26 ± 4.64 | 24.82 ± 4.87 | 21.64 ± 2.03 | 23.76 ± 1.35 | 0.015 | 0.305 | 0.305 | 0.056 |
| **Time since injury** (months) | 12.29 ± 0.77 | 13.10 ± 1.67 | 12.63 ± 1.15 | 12.30 ± 0.95 | -- | -- | < 0.0001 | -- | 0.579 | -- |
| **Sex ♀:♂** (%) | 17:21 (44.7:55.3) | | 16:10 (61.5:38.5) | | 20:18 (52.6:47.4) | | 0.494 | 0.848 | 0.19 | 0.419 |
| **Leg dominance** (right:left) | 16:1 | 19:2 | 15:1 | 8:2 | 19:1 | 15:3 | 0.694 | 0.899 | 0.626 | 0.876 |
| **Prehabilitation** (yes:no) (% of subgroup) | 8:9 (47.1:52.9) | 5:16  (23.8:76.2) | -- | -- | -- | -- | -- | -- | -- | -- |
| **Prehabilitation** (yes:no)  (% of subgroup) | 13:25  (34.2:65.8) | | -- | | -- | | < 0.0001 | -- | < 0.0001 | -- |
| **PT after surgery/injury** (yes:no) | 17:0 | 21:0 | 16:0 | 10:0 | -- | -- | -- | -- | -- | -- |
| **PT after surgery/injury** (yes:no) | 38:0 | | 26:0 | | -- | | < 0.0001 | -- | < 0.0001 | -- |
| **Physical activity** [min/week] | 447.68 ± 213.66 | 408.38 ± 304.58 | 362.41 ± 168.87 | 390.00 ± 146.46 | 293.80 ± 213.21 | 292.67 ± 148.10 | 0.014 | 0.011 | 0.811 | 0.014 |
| **Tegner score** (max. 10 points)° | 6.53 ± 1.55 | 6.86 ± 1.39 | 6.56 ± 0.81 | 7.60 ± 1.43 | 5.40 ± 1.54 | 5.67 ± 1.03 | 0.001 | < 0.0001 | 0.273 | < 0.0001 |
| **KOOS subscale (absolute values)**  pain (9 items, max. 36 p.)  other symptoms (7 items, max. 28 p.)  ADL (17 items, max. 68 p.)  sports & leisure (5 items, max. 20 p.)  HRQoL (4 items, max. 16 p.) | 31.82 ± 3.30  21.94 ± 2.75  65.18 ± 3.70  16.12 ± 3.02  12.00 ± 2.81 | 31.29 ± 3.40  22.14 ± 4.54  65.76 ± 3.24  16.57 ±2.82  10.67 ± 3.58 | 32.63 ± 3.40  22.70 ± 3.42  65.25 ± 4.77  17.06 ± 3.07  11.63 ± 3.12 | 32.70 ± 2.36  23.30 ± 2.71  66.60 ±2.46  17.70 ± 2.31  13.10 ± 2.38 | 35.85 ± 0.49  26.40 ± 1.31  67.90 ± 0.45  19.95 ± 0.22  15.70 ± 0.92 | 35.44 ± 0.86  26.44 ± 1.76  67.94 ± 0.24  19.61 ± 0.98  15.56 ± 1.10 | < 0.0001  < 0.0001  < 0.0001  < 0.0001  < 0.0001 | < 0.0001  < 0.0001  < 0.0001  < 0.0001  < 0.0001 | 0.119  0.532  0.380  0.151  0.259 | < 0.0001  < 0.0001  < 0.0001  < 0.0001  < 0.0001 |
| **VAS**  wellbeing pre [mm]  wellbeing post [mm]  pain pre [mm]  pain post [mm] | 6.12 ± 10.27  6.71 ± 10.69  3.41 ± 5.23  6.65 ± 11.26 | 5.05 ± 7.21  7.76 ± 7.60  2.81 ± 2.94  5.81 ± 14.94 | 7.94 ± 12.50  8.13 ± 11.93  6.25 ± 10.93  9.25 ± 12.64 | 1.20 ± 1.99  1.50 ± 2.42  1.30 ± 1.95  1.90 ± 2.73 | 4.50 ± 5.48  6.85 ± 8.11  0.95 ± 1.76  4.75 ± 10.44 | 6.17 ± 8.28  6.22 ± 6.72  1.22 ± 2.39  1.94 ± 3.02 | 0.643  0.937  0.026  0.264 | 0.666  0.355  0.037  0.082 | 0.972  0.402  0.977  0.486 | 0.870  0.612  0.045  0.209 |
| **Medial meniscal tears** (N=)  conservative treatment  suture  resection | 0  7  1 | 2  8  6 | 3  0  0 | 1  0  0 | --  --  -- | --  --  -- | --  --  -- | --  --  -- | --  --  -- | --  --  -- |
| **Lateral meniscal tears** (N=)  conservative treatment  suture  resection | 1  3  0 | 0  5  1 | 4  0  0 | 1  0  0 | --  --  -- | --  --  -- | --  --  -- | --  --  -- | --  --  -- | --  --  -- |
| **Medial collateral ligament injury** (N=)  conservative treatment  surgery | 3  1 | 5  0 | 5  0 | 3  0 | --  -- | --  -- | --  -- | --  -- | --  -- | --  -- |
| **Lateral collateral ligament injury** (N=)  conservative treatment  surgery | 1  0 | 1  0 | 3  0 | 1  0 | --  -- | --  -- | --  -- | --  -- | --  -- | --  -- |
| **Bone bruise**  (N=) (% of subgroup) | 0 (0) | 0 (0) | 6 (37.5) | 1 (6.3) | -- | -- | -- | -- | -- | -- |
| **Cartilage defect**  (N=) (% of subgroup) | 0 (0) | 2 (9.5) | 2 (12.5) | 0 (0) | -- | -- | -- | -- | -- | -- |
| **Graft types** (N=) (%)  Quadriceps tendon  Hamstrings tendon  Patellar tendon  Unknown | 10 (58.8)  5 (29.4)  1 (5.9)  1 (5.9) | 16 (76.2)  3 (14.3)  2 (9.5)  0 (0) | --  --  --  -- | --  --  --  -- | --  --  --  -- | --  --  --  -- | --  --  --  -- | --  --  --  -- | --  --  --  -- | --  --  --  -- |

If not otherwise stated means, ± standard deviations and p-values are reported. Dashed lines indicate not applicable.

Legend: ACL-R = anterior cruciate ligament reconstructed (=patients); ACL-C = anterior cruciate ligament rupture conservatively treated; ACL-I = anterior cruciate ligament intact (= healthy controls); KOOS = Knee injury and Osteoarthritis Outcome Score; ADL = activity of daily life; HRQoL = health-related quality of life; N= number of; pre = before the measurements started; PT = physiotherapy; post = after the measurements; VAS = visual analogue scale from 0 to 100mm; % = percentage of subgroup; °Tegner activity score (preinjury) ranging from 0 (sick leave or disability pension) to 10 (competitive sport on a professional level)
