# Supplementary material for: Neuromuscular control in males and females 1 year after an anterior cruciate ligament rupture or reconstruction during stair descent and artificial tibial translation
Source: Sci Rep. 2023 Sep 15;13:15316. doi: 10.1038/s41598-023-42491-6 (PMC10504317; doi:10.1038/s41598-023-42491-6)
Supplement: Supplementary file 3 — SupplementaryTable 3. [file 41598_2023_42491_MOESM3_ESM.docx]

Table A.3: Stair descent: Neuromuscular activity for females and males per group for the involved (injured), matched limb respectively

| **Muscle** | **Group** | | | | | | **p-values** | | | | | | | **Effect size** | | | | | |
| --- | --- | --- | --- | --- | --- | --- | --- | --- | --- | --- | --- | --- | --- | --- | --- | --- | --- | --- | --- |
|  | **ACL-R** | | **ACL-C** | | **ACL-I = Control** | |  |  |  |  |  |  |  |  |  |  |  |  |  |
|  | females | males | females | males | females | males | overall* | [1]vs[3]° | [1]vs[5]° | [3]vs[5]° | [2]vs[4]° | [2]vs[6]° | [4]vs[6]° | [1]vs[3]° | [1]vs[5]° | [3]vs[5]° | [2]vs[4]° | [2]vs[6]° | [4]vs[6]° |
|  | [1] | [2] | [3] | [4] | [5] | [6] |  |  |  |  |  |  |  |  |  |  |  |  |  |
| **VM** | 131.8 (38.9) | 125.6 (32.1) | 116.1 (31.9) | 118.2 (41.5) | 117.5 (38.4) | 110.5 (42.5) | 0.328 | 0.234 | 0.448 | 0.745 | 0.538 | 0.273 | 0.688 | -- | -- | -- | -- | -- | -- |
|  |  |  |  |  |  |  |  |  |  |  |  |  |  |  |  |  |  |  |  |
| **VL** | 126.3 (26.1) | 124.6 (28.8) | 127.4 (24.2) | 118.6 (24.3) | 133.3 (44.1) | 108.1 (41.0) | 0.806 | 0.865 | 0.503 | 0.617 | 0.598 | 0.094 | 0.482 | -- | -- | -- | -- | -- | -- |
|  |  |  |  |  |  |  |  |  |  |  |  |  |  |  |  |  |  |  |  |
| **BF** | 96.8 (36.7) | 100.5 (37.0) | 115.3 (43.7) | 90.8 (40.5) | 117.4 (45.3) | 108.9 (43.1) | 0.362 | 0.167 | 0.134 | 0.975 | 0.735 | 0.693 | 0.388 | -- | -- | -- | -- | -- | -- |
|  |  |  |  |  |  |  |  |  |  |  |  |  |  |  |  |  |  |  |  |
| **ST** | 102.3 (34.5) | 96.8 (39.7) | 107.7 (33.7) | 107.8 (42.0) | 124.6 (40.4) | 114.1 (52.3) | 0.174 | 0.64 | 0.106 | 0.121 | 0.605 | 0.274 | 0.647 | -- | -- | -- | -- | -- | -- |
|  |  |  |  |  |  |  |  |  |  |  |  |  |  |  |  |  |  |  |  |
| **Stair descent, weight acceptance, involved/matched side** | | | | | | | | | | | | | | | | | | | |
| **Muscle** | **Group** | | | | | | **p-values** | | | | | | | **Effect size** | | | | | |
|  | **ACL-R** | | **ACL-C** | | **ACL-I = Control** | |  |  |  |  |  |  |  |  |  |  |  |  |  |
|  | females | males | females | males | females | males | overall* | [1]vs[3]° | [1]vs[5]° | [3]vs[5]° | [2]vs[4]° | [2]vs[6]° | [4]vs[6]° | [1]vs[3]° | [1]vs[5]° | [3]vs[5]° | [2]vs[4]° | [2]vs[6]° | [4]vs[6]° |
|  | [1] | [2] | [3] | [4] | [5] | [6] |  |  |  |  |  |  |  |  |  |  |  |  |  |
| **VM** | 274.9 (76.3) | 251.4 (105.0) | 213.9 (75.6) | 254.0 (50.1) | 274.3 (104.4) | 252.0 (136.2) | 0.654 | **0.038** | 0.951 | 0.083 | 0.235 | 0.626 | 0.451 | -- | -- | -- | -- | -- | -- |
|  |  |  |  |  |  |  |  |  |  |  |  |  |  |  |  |  |  |  |  |
| **VL** | 258.6 (60.0) | 238.6 (75.8) | 243.2 (53.7) | 152.3 (46.2) | 250.8 (86.6) | 201.7 (77.5) | 0.331 | 0.234 | 0.670 | 0.806 | 0.356 | 0.135 | **0.031** | -- | -- | -- | -- | -- | -- |
|  |  |  |  |  |  |  |  |  |  |  |  |  |  |  |  |  |  |  |  |
| **BF** | 73.1 (30.9) | 77.6 (26.8) | 59.2 (22.6) | 55.2 (19.3) | 82.2 (61.4) | 91.4 (50.2) | 0.051 | 0.117 | 0.831 | 0.790 | **0.039** | 0.503 | 0.056 | -- | -- | -- | -- | -- | -- |
|  |  |  |  |  |  |  |  |  |  |  |  |  |  |  |  |  |  |  |  |
| **ST** | 40.1 (14.8) | 51.4 (21.9) | 51.6 (21.7) | 38.1 (19.2) | 59.8 (39.6) | 54.6 (35.0) | 0.693 | 0.114 | 0.136 | 0.959 | 0.215 | 0.716 | 0.317 | -- | -- | -- | -- | -- | -- |
|  |  |  |  |  |  |  |  |  |  |  |  |  |  |  |  |  |  |  |  |
| **Stair descent, push-off, involved/matched side** | | | | | | | | | | | | | | | | | | | |
| **Muscle** | **Group** | | | | | | **p-values** | | | | | | | **Effect size** | | | | | |
|  | **ACL-R** | | **ACL-C** | | **ACL-I = Control** | |  |  |  |  |  |  |  |  |  |  |  |  |  |
|  | females | males | females | males | females | males | overall* | [1]vs[3]° | [1]vs[5]° | [3]vs[5]° | [2]vs[4]° | [2]vs[6]° | [4]vs[6]° | [1]vs[3]° | [1]vs[5]° | [3]vs[5]° | [2]vs[4]° | [2]vs[6]° | [4]vs[6]° |
|  | [1] | [2] | [3] | [4] | [5] | [6] |  |  |  |  |  |  |  |  |  |  |  |  |  |
| **VM** | 336.4 (145.8) | 251.9 (118.0) | 269.5 (143.2) | 414.8 (115.2) | 262.2 (123.7) | 207.9 (92.6) | 0.081 | 0.097 | 0.233 | 0.665 | **0.002** | 0.273 | **< 0.001** | -- | -- | -- | -- | -- | -- |
|  |  |  |  |  |  |  |  |  |  |  |  |  |  |  |  |  |  |  |  |
| **VL** | 297.0 (95.4) | 216.8 (104.2) | 216.9 (70.5) | 309.3 (65.8) | 236.0 (100.4) | 162.6 (79.4) | 0.069 | **0.043** | 0.211 | 0.345 | **0.014** | 0.135 | **< 0.001** | -- | -- | -- | -- | -- | -- |
|  |  |  |  |  |  |  |  |  |  |  |  |  |  |  |  |  |  |  |  |
| **BF** | 49.0 (17.2) | 48.4 (19.7) | 57.6 (26.9) | 37.9 (18.2) | 76.0 (36.9) | 82.1 (48.3) | **0.001** | 0.777 | 0.055 | 0.131 | 0.218 | **0.009** | **0.007** | -- | -- | -- | -- | 0.43 | 0.52 |
|  |  |  |  |  |  |  |  |  |  |  |  |  |  |  |  |  |  |  |  |
| **ST** | 52.1 (19.7) | 52.9 (26.2) | 65.3 (24.4) | 47.5 (29.6) | 70.4 (33.1) | 59.0 (22.3) | 0.146 | 0.136 | 0.084 | 0.822 | 0.628 | 0.265 | 0.14 | -- | -- | -- | -- | -- | -- |
|  |  |  |  |  |  |  |  |  |  |  |  |  |  |  |  |  |  |  |  |

Legend: Normalized root mean square (RMS) values, expressed as % of submaximal voluntary contraction (during treadmill walking), are reported per muscle and movement phase during stair descent. If not otherwise stated means, standard deviations (in brackets) and p-values are reported. *Kruskal-Wallis test; °Mann-Whitney-U test. Boldface **p-values** indicate statistically significant differences between subgroups (p<0.05). Dashes indicate not applicable. ACL = anterior cruciate ligament; ACL-R = anterior cruciate ligament reconstructed (=patients); ACL-C = anterior cruciate ligament rupture conservatively treated; ACL-I = anterior cruciate ligament intact (= healthy controls); BF = biceps femoris; involved = injured leg, respective matched leg of controls (based on side of injury); PO = push-off; PRE = pre-activity; RMS = root mean square; SD = standard deviation; ST = semitendinosus; VM = vastus medialis; VL = vastus lateralis; WA = weight acceptance
