# Supplementary material for: Neuromuscular control in males and females 1 year after an anterior cruciate ligament rupture or reconstruction during stair descent and artificial tibial translation
Source: Sci Rep. 2023 Sep 15;13:15316. doi: 10.1038/s41598-023-42491-6 (PMC10504317; doi:10.1038/s41598-023-42491-6)
Supplement: Supplementary file 4 — Supplementary Table 4. [file 41598_2023_42491_MOESM4_ESM.docx]

Table A.4: Artificial tibial translation: Reflex activity for females and males per group per phase for the involved (injured), matched limb respectively

| **Reflex activity, pre-activation 50ms (PRE_50), involved/matched side** | | | | | | | | | | | | | | | | | | | |
| --- | --- | --- | --- | --- | --- | --- | --- | --- | --- | --- | --- | --- | --- | --- | --- | --- | --- | --- | --- |
| **Muscle** | **Group** | | | | | | **p-values** | | | | | | | **Effect size** | | | | | |
|  | **ACL-R** | | **ACL-C** | | **ACL-I = Control** | |  |  |  |  |  |  |  |  |  |  |  |  |  |
|  | females | males | females | males | females | males | overall* | [1]vs[3]° | [1]vs[5]° | [3]vs[5]° | [2]vs[4]° | [2]vs[6]° | [4]vs[6]° | [1]vs[3]° | [1]vs[5]° | [3]vs[5]° | [2]vs[4]° | [2]vs[6]° | [4]vs[6]° |
|  | [1] | [2] | [3] | [4] | [5] | [6] |  |  |  |  |  |  |  |  |  |  |  |  |  |
| **VM** | 243.6 (288.3) | 127.7 (57.1) | 111.2 (39.1) | 85.1 (28.2) | 153.0 (66.7) | 125.3 (66.5) | **0.013** | **0.018** | 0.466 | **0.034** | **0.025** | 0.599 | 0.164 | 0.43 | -- | 0.36 | 0.41 | -- | -- |
|  |  |  |  |  |  |  |  |  |  |  |  |  |  |  |  |  |  |  |  |
| **VL** | 199.7 (138.0) | 126.5 (60.3) | 126.0 (33.1) | 117.7 (39.5) | 152.4 (49.9) | 140.6 (64.1) | 0.229 | **0.010** | 0.229 | 0.120 | 0.966 | 0.297 | 0.362 | -- | -- | -- | -- | -- | -- |
|  |  |  |  |  |  |  |  |  |  |  |  |  |  |  |  |  |  |  |  |
| **BF** | 56.8 (52.5) | 41.1 (22.4) | 43.3 (28.1) | 40.4 (28.3) | 53.9 (39.0) | 79.1 (40.6) | **0.035** | 0.827 | 0.636 | 0.435 | 0.860 | **0.003** | **0.019** | -- | -- | -- | -- | 0.48 | 0.44 |
|  |  |  |  |  |  |  |  |  |  |  |  |  |  |  |  |  |  |  |  |
| **ST** | 57.3 (21.1) | 62.6 (34.6) | 62.6 (27.5) | 54.8 (31.6) | 56.5 (30.8) | 35.4 (29.1) | 0.099 | 0.647 | 0.716 | 0.716 | 0.538 | **0.009** | 0.084 | -- | -- | -- | -- | -- | -- |
|  |  |  |  |  |  |  |  |  |  |  |  |  |  |  |  |  |  |  |  |
| **Reflex activity, short latency response (SLR), involved/matched side** | | | | | | | | | | | | | | | | | | | |
| **Muscle** | **Group** | | | | | | **p-values** | | | | | | | **Effect size** | | | | | |
|  | **ACL-R** | | **ACL-C** | | **ACL-I = Control** | |  |  |  |  |  |  |  |  |  |  |  |  |  |
|  | females | males | females | males | females | males | overall* | [1]vs[3]° | [1]vs[5]° | [3]vs[5]° | [2]vs[4]° | [2]vs[6]° | [4]vs[6]° | [1]vs[3]° | [1]vs[5]° | [3]vs[5]° | [2]vs[4]° | [2]vs[6]° | [4]vs[6]° |
|  | [1] | [2] | [3] | [4] | [5] | [6] |  |  |  |  |  |  |  |  |  |  |  |  |  |
| **VM** | 377.7 (289.1) | 181.0 (114.4) | 202.6 (131.8) | 181.8 (140.3) | 205.0 (75.8) | 177.1 (128.3) | 0.582 | 0.088 | 0.126 | 0.667 | 0.692 | 0.661 | 0.962 | -- | -- | -- | -- | -- | -- |
|  |  |  |  |  |  |  |  |  |  |  |  |  |  |  |  |  |  |  |  |
| **VL** | 315.1 (149.4) | 139.5 (65.5) | 178.1 (101.4) | 159.4 (69.1) | 170.4 (48.4) | 162.3 (96.5) | 0.643 | **0.007** | **0.003** | 0.817 | 0.402 | 0.509 | 0.808 | -- | -- | -- | -- | -- | -- |
|  |  |  |  |  |  |  |  |  |  |  |  |  |  |  |  |  |  |  |  |
| **BF** | 292.9 (442.0) | 237.0 (327.4) | 215.8 (136.7) | 172.6 (153.7) | 192.7 (124.6) | 220.3 (166.9) | 0.570 | 0.570 | 0.732 | 0.800 | 0.860 | 0.279 | 0.231 | -- | -- | -- | -- | -- | -- |
|  |  |  |  |  |  |  |  |  |  |  |  |  |  |  |  |  |  |  |  |
| **ST** | 215.4 (204.7) | 256.3 (238.6) | 219.9 (181.2) | 185.2 (141.1) | 272.3 (343.0) | 189.4 (259.4) | 0.351 | 0.694 | 0.799 | 0.544 | 0.455 | 0.077 | 0.366 | -- | -- | -- | -- | -- | -- |
|  |  |  |  |  |  |  |  |  |  |  |  |  |  |  |  |  |  |  |  |
| **Reflex activity, medium latency response (MLR), involved/matched side** | | | | | | | | | | | | | | | | | | | |
| **Muscle** | **Group** | | | | | | **p-values** | | | | | | | **Effect size** | | | | | |
|  | **ACL-R** | | **ACL-C** | | **ACL-I = Control** | |  |  |  |  |  |  |  |  |  |  |  |  |  |
|  | females | males | females | males | females | males | overall* | [1]vs[3]° | [1]vs[5]° | [3]vs[5]° | [2]vs[4]° | [2]vs[6]° | [4]vs[6]° | [1]vs[3]° | [1]vs[5]° | [3]vs[5]° | [2]vs[4]° | [2]vs[6]° | [4]vs[6]° |
|  | [1] | [2] | [3] | [4] | [5] | [6] |  |  |  |  |  |  |  |  |  |  |  |  |  |
| **VM** | 516.6 (319.9) | 372.4 (461.1) | 239.0 (98.5) | 313.3 (207.2) | 340.7 (155.2) | 317.7 (202.8) | 0.097 | **0.001** | 0.117 | **0.040** | 0.888 | 0.599 | 1.000 | -- | -- | -- | -- | -- | -- |
|  |  |  |  |  |  |  |  |  |  |  |  |  |  |  |  |  |  |  |  |
| **VL** | 511.8 (279.9) | 300.7 (186.0) | 301.4 (128.5) | 298.8 (128.9) | 305.3 (133.3) | 321.3 (196.0) | 0.472 | **0.031** | **0.028** | 0.945 | 0.769 | 0.735 | 0.797 | -- | -- | -- | -- | -- | -- |
|  |  |  |  |  |  |  |  |  |  |  |  |  |  |  |  |  |  |  |  |
| **BF** | 294.9 (190.3) | 234.3 (213.7) | 268.8 (147.4) | 252.3 (127.7) | 391.3 (347.1) | 362.4 (280.6) | 0.325 | 0.792 | 0.687 | 0.551 | 0.567 | 0.152 | 0.472 | -- | -- | -- | -- | -- | -- |
|  |  |  |  |  |  |  |  |  |  |  |  |  |  |  |  |  |  |  |  |
| **ST** | 180.3 (120.5) | 208.8 (213.7) | 216.9 (143.6) | 208.4 (87.3) | 500.3 (499.4) | 418.1 (408.9) | **0.011** | 0.475 | **0.015** | 0.042 | 0.312 | 0.161 | 0.422 | -- | 0.43 | -- | -- | -- | -- |
|  |  |  |  |  |  |  |  |  |  |  |  |  |  |  |  |  |  |  |  |
| **Reflex activity, long latency response (LLR), involved/matched side** | | | | | | | | | | | | | | | | | | | |
| **Muscle** | **Group** | | | | | | **p-values** | | | | | | | **Effect size** | | | | | |
|  | **ACL-R** | | **ACL-C** | | **ACL-I = Control** | |  |  |  |  |  |  |  |  |  |  |  |  |  |
|  | females | males | females | males | females | males | overall* | [1]vs[3]° | [1]vs[5]° | [3]vs[5]° | [2]vs[4]° | [2]vs[6]° | [4]vs[6]° | [1]vs[3]° | [1]vs[5]° | [3]vs[5]° | [2]vs[4]° | [2]vs[6]° | [4]vs[6]° |
|  | [1] | [2] | [3] | [4] | [5] | [6] |  |  |  |  |  |  |  |  |  |  |  |  |  |
| **VM** | 435.1 (368.1) | 387.5 (553.5) | 212.3 (145.0) | 233.7 (166.9) | 238.3 (139.7) | 186.9 (142.6) | **0.034** | **0.015** | **0.049** | 0.591 | 0.538 | 0.075 | 0.388 | 0.45 | 0.34 | -- | -- | -- | -- |
|  |  |  |  |  |  |  |  |  |  |  |  |  |  |  |  |  |  |  |  |
| **VL** | 311.5 (162.7) | 272.4 (203.3) | 258.3 (180.7) | 199.9 (122.9) | 233.7 (129.9) | 174.9 (102.5) | 0.110 | 0.313 | 0.199 | 0.947 | 0.272 | 0.055 | 0.632 | -- | -- | -- | -- | -- | -- |
|  |  |  |  |  |  |  |  |  |  |  |  |  |  |  |  |  |  |  |  |
| **BF** | 233.7 (118.3) | 186.4 (134.1) | 225.0 (114.6) | 233.3 (127.9) | 230.0 (139.5) | 266.0 (112.1) | 0.624 | 0.730 | 0.841 | 0.885 | 0.272 | 0.215 | 0.848 | -- | -- | -- | -- | -- | -- |
|  |  |  |  |  |  |  |  |  |  |  |  |  |  |  |  |  |  |  |  |
| **ST** | 233.1 (132.9) | 267.9 (265.0) | 260.6 (190.8) | 379.3 (231.7) | 225.7 (144.7) | 227.5 (178.7) | 0.457 | 0.930 | 0.893 | 0.716 | 0.190 | 0.941 | 0.108 | -- | -- | -- | -- | -- | -- |
|  |  |  |  |  |  |  |  |  |  |  |  |  |  |  |  |  |  |  |  |

Legend: Normalized root mean square (RMS) values, expressed as % of submaximal voluntary contraction (during treadmill walking), are reported per muscle and reflex window. If not otherwise stated means, standard deviations (in brackets) and p-values are reported. *Kruskal-Wallis test; °Mann-Whitney-U test. Boldface **p-values** indicate statistically significant differences between subgroups (p<0.05). Dashes indicate not applicable. ACL = anterior cruciate ligament; ACL-R = anterior cruciate ligament reconstructed (=patients); ACL-C = anterior cruciate ligament rupture conservatively treated; ACL-I = anterior cruciate ligament intact (= healthy controls); BF = biceps femoris; involved = injured leg, respective matched leg of controls (based on side of injury); LLR = long latency response; MLR = medium latency response; PRE_50 = pre-activity; SLR = short latency response; ST = semitendinosus; VM = vastus medialis; VL = vastus lateralis
