# Supplementary material for: Neuromuscular control in males and females 1 year after an anterior cruciate ligament rupture or reconstruction during stair descent and artificial tibial translation
Source: Sci Rep. 2023 Sep 15;13:15316. doi: 10.1038/s41598-023-42491-6 (PMC10504317; doi:10.1038/s41598-023-42491-6)
Supplement: Supplementary file 5 — Supplementary Table 5. [file 41598_2023_42491_MOESM5_ESM.docx]

Table A.5a: Tests of between-subjects effects by two-way parametric ANOVA (significant results for involved side)

| **Factor** | **Dependent variable** | **Typ III sum of squares** | **df** | **Means of squares** | **F** | **Sig.** | **Partial eta square** |
| --- | --- | --- | --- | --- | --- | --- | --- |
| group | PO of BF during stair descent | 15557.87 | 2 | 7778.93 | 9.191 | < 0.0001 | 0.183 |
|  | PRE_50 of VM during reflex measurements | 105944.80 | 2 | 52972.40 | 3.471 | 0.036 | 0.078 |
|  | PRE_50 of BF during reflex measurements | 9052.15 | 2 | 4526.08 | 3.840 | 0.025 | 0.086 |
|  | MLR of ST during reflex measurements | 1536569.94 | 2 | 768284.97 | 8.020 | 0.001 | 0.164 |
|  | LLR of VM for reflex measurements | 339665.53 | 2 | 169832.76 | 2.104 | 0.129 | 0.049 |
| sex | PO of BF during stair descent | 1010.30 | 1 | 1010.30 | 1.194 | 0.278 | 0.014 |
|  | PRE_50 of VM during reflex measurements | 97430.24 | 1 | 97430.24 | 6.385 | 0.013 | 0.072 |
|  | PRE_50 of BF during reflex measurements | 326.36 | 1 | 326.36 | 0.277 | 0.600 | 0.003 |
|  | MLR of ST during reflex measurements | 17664.55 | 1 | 17664.55 | 0.184 | 0.669 | 0.002 |
|  | LLR of VM for reflex measurements | 5858.91 | 1 | 5858.91 | 0.073 | 0.788 | 0.001 |
| group * sex | PO of BF during stair descent | 1911.09 | 2 | 955.55 | 1.129 | 0.328 | 0.027 |
|  | PRE_50 of VM during reflex measurements | 54267.71 | 2 | 27133.85 | 1.778 | 0.175 | 0.042 |
|  | PRE_50 of BF during reflex measurements | 4727.81 | 2 | 2363.91 | 2.006 | 0.141 | 0.047 |
|  | MLR of ST during reflex measurements | 20500.00 | 2 | 10250.00 | 0.107 | 0.899 | 0.003 |
|  | LLR of VM for reflex measurements | 32076.96 | 2 | 16038.48 | 0.199 | 0.820 | 0.005 |

Legend: BF = biceps femoris; df = degrees of freedom; F = F-value; involved side = injured leg, respective matched leg of controls (based on side of injury); LLR = long latency response; MLR = medium latency; PO = push-off; PRE_50 = pre-activation (-50-0 ms background activity); Sig. = significance (p-value); ST = semitendinosus; VM = vastus medialis

Table A.5b: Stair descent: Neuromuscular activity for females and males per group, including values for calculation of effect sizes (for significant results)

| **Stair descent, push-off, involved/matched side** | | | | | | | | | | | | | | | | | | | |
| --- | --- | --- | --- | --- | --- | --- | --- | --- | --- | --- | --- | --- | --- | --- | --- | --- | --- | --- | --- |
| **Muscle** | **Group** | | | | | | **p-values** | | | | | | | **Effect size (Z-value) *N=*** | | | | | |
|  | **ACL-R** | | **ACL-C** | | **ACL-I = Control** | |  |  |  |  |  |  |  |  |  |  |  |  |  |
|  | females | males | females | males | females | males | overall* | [1]vs[3]° | [1]vs[5]° | [3]vs[5]° | [2]vs[4]° | [2]vs[6]° | [4]vs[6]° | [1]vs[3]° | [1]vs[5]° | [3]vs[5]° | [2]vs[4]° | [2]vs[6]° | [4]vs[6]° |
|  | [1] | [2] | [3] | [4] | [5] | [6] |  |  |  |  |  |  |  |  |  |  |  |  |  |
| **BF** | 49.0 (17.2) | 48.4 (19.7) | 57.6 (26.9) | 37.9 (18.2) | 76.0 (36.9) | 82.1 (48.3) | **0.001** | 0.777 | 0.055 | 0.131 | 0.218 | **0.009** | **0.007** | -- | -- | -- | -- | 0.43  (-2,621)  *N=37* | 0.52  (-2.711)  *N=27* |

Legend: Normalized root mean square (RMS) values, expressed as % of submaximal voluntary contraction (during treadmill walking), are reported per muscle and movement phase during stair descent. If not otherwise stated means, standard deviations (in brackets) and p-values are reported. *Kruskal-Wallis test; °Mann-Whitney-U test. Boldface **p-values** indicate statistically significant differences between subgroups (p<0.05). Dashes indicate not applicable. ACL = anterior cruciate ligament; ACL-R = anterior cruciate ligament reconstructed (=patients); ACL-C = anterior cruciate ligament rupture conservatively treated; ACL-I = anterior cruciate ligament intact (= healthy controls); BF = biceps femoris; involved = injured leg, respective matched leg of controls (based on side of injury); N= number of ; RMS = root mean square; SD = standard deviation

Table A.5c: Reflex activity: Neuromuscular activity for females and males per group, including values for calculation of effect sizes (for significant results)

| **Reflex activity, pre-activation 50ms (PRE_50), involved/matched side** | | | | | | | | | | | | | | | | | | | |
| --- | --- | --- | --- | --- | --- | --- | --- | --- | --- | --- | --- | --- | --- | --- | --- | --- | --- | --- | --- |
| **Muscle** | **Group** | | | | | | **p-values** | | | | | | | **Effect size (Z-value) *N=*** | | | | | |
|  | **ACL-R** | | **ACL-C** | | **ACL-I = Control** | |  |  |  |  |  |  |  |  |  |  |  |  |  |
|  | females | males | females | males | females | males | overall* | [1]vs[3]° | [1]vs[5]° | [3]vs[5]° | [2]vs[4]° | [2]vs[6]° | [4]vs[6]° | [1]vs[3]° | [1]vs[5]° | [3]vs[5]° | [2]vs[4]° | [2]vs[6]° | [4]vs[6]° |
|  | [1] | [2] | [3] | [4] | [5] | [6] |  |  |  |  |  |  |  |  |  |  |  |  |  |
| **VM** | 243.6 (288.3) | 127.7 (57.1) | 111.2 (39.1) | 85.1 (28.2) | 153.0 (66.7) | 125.3 (66.5) | **0.013** | **0.018** | 0.466 | **0.034** | **0.025** | 0.599 | 0.164 | 0.43  (-2,370)  *N=30* | -- | 0.36  (-2,119)  *N=35* | 0.41  (-2.244)  *N=30* | -- | -- |
| **BF** | 56.8 (52.5) | 41.1 (22.4) | 43.3 (28.1) | 40.4 (28.3) | 53.9 (39.0) | 79.1 (40.6) | **0.035** | 0.827 | 0.636 | 0.435 | 0.860 | **0.003** | **0.019** | -- | -- | -- | -- | 0.48  (-2,953)  *N=38* | 0.44  (-2,349)  *N=28* |
| **Reflex activity, medium latency response (MLR), involved/matched side** | | | | | | | | | | | | | | | | | | | |
| **Muscle** | **Group** | | | | | | **p-values** | | | | | | | **Effect size (Z-value) *N=*** | | | | | |
|  | **ACL-R** | | **ACL-C** | | **ACL-I = Control** | |  |  |  |  |  |  |  |  |  |  |  |  |  |
|  | females | males | females | males | females | males | overall* | [1]vs[3]° | [1]vs[5]° | [3]vs[5]° | [2]vs[4]° | [2]vs[6]° | [4]vs[6]° | [1]vs[3]° | [1]vs[5]° | [3]vs[5]° | [2]vs[4]° | [2]vs[6]° | [4]vs[6]° |
|  | [1] | [2] | [3] | [4] | [5] | [6] |  |  |  |  |  |  |  |  |  |  |  |  |  |
| **ST** | 180.3 (120.5) | 208.8 (213.7) | 216.9 (143.6) | 208.4 (87.3) | 500.3 (499.4) | 418.1 (408.9) | **0.011** | 0.475 | **0.015** | 0.042 | 0.312 | 0.161 | 0.422 | -- | 0.43  (-2,436)  *N=32* | -- | -- | -- | -- |
| **Reflex activity, long latency response (LLR), involved/matched side** | | | | | | | | | | | | | | | | | | | |
| **Muscle** | **Group** | | | | | | **p-values** | | | | | | | **Effect size (Z-value) *N=*** | | | | | |
|  | **ACL-R** | | **ACL-C** | | **ACL-I = Control** | |  |  |  |  |  |  |  |  |  |  |  |  |  |
|  | females | males | females | males | females | males | overall* | [1]vs[3]° | [1]vs[5]° | [3]vs[5]° | [2]vs[4]° | [2]vs[6]° | [4]vs[6]° | [1]vs[3]° | [1]vs[5]° | [3]vs[5]° | [2]vs[4]° | [2]vs[6]° | [4]vs[6]° |
|  | [1] | [2] | [3] | [4] | [5] | [6] |  |  |  |  |  |  |  |  |  |  |  |  |  |
| **VM** | 435.1 (368.1) | 387.5 (553.5) | 212.3 (145.0) | 233.7 (166.9) | 238.3 (139.7) | 186.9 (142.6) | **0.034** | **0.015** | **0.049** | 0.591 | 0.538 | 0.075 | 0.388 | 0.45  (-2,444)  *N=29* | 0.34  (-1,967)  *N=33* | -- | -- | -- | -- |

Legend: Normalized root mean square (RMS) values, expressed as % of submaximal voluntary contraction (during treadmill walking), are reported per muscle and reflex window. If not otherwise stated means, standard deviations (in brackets) and p-values are reported. *Kruskal-Wallis test; °Mann-Whitney-U test. Boldface **p-values** indicate statistically significant differences between subgroups (p<0.05). Dashes indicate not applicable. ACL = anterior cruciate ligament; ACL-R = anterior cruciate ligament reconstructed (=patients); ACL-C = anterior cruciate ligament rupture conservatively treated; ACL-I = anterior cruciate ligament intact (= healthy controls); BF = biceps femoris; involved = injured leg, respective matched leg of controls (based on side of injury); LLR = long latency response; MLR = medium latency response; PRE_50 = pre-activity; ST = semitendinosus; VM = vastus medialis
